# Design and development of a sensorized hammerstone for accurate force measurement in stone knapping experiments

**DOI:** 10.1371/journal.pone.0310520

**Published:** 2024-09-17

**Authors:** Cecilia Barroso-Medina, Sam C. Lin, Matthew W. Tocheri, Manish Sreenivasa

**Affiliations:** 1 School of Earth, Atmospheric and Life Sciences, University of Wollongong, Wollongong, NSW, Australia; 2 Australian Research Council Centre of Excellence for Australian Biodiversity and Heritage, University of Wollongong, Wollongong, NSW, Australia; 3 Department of Anthropology, Lakehead University, Thunder Bay, Ontario, Canada; 4 Human Origins Program, National Museum of Natural History, Smithsonian Institution, Washington, D.C., United States of America; 5 School of Mechanical, Materials, Mechatronic and Biomedical Engineering, University of Wollongong, Wollongong, NSW, Australia; Sapienza University of Rome: Universita degli Studi di Roma La Sapienza, ITALY

## Abstract

The process of making stone tools, specifically knapping, is a hominin behaviour that typically involves using the upper limb to manipulate a stone hammer and apply concentrated percussive force to another stone, causing fracture and detachment of stone chips with sharp edges. To understand the emergence and subsequent evolution of tool-related behaviours in hominins, the connections between the mechanics of stone knapping, including the delivery of percussive forces, and biomechanics and hominin anatomy, especially in the upper limb, are required. However, there is an absence of direct experimental means to measure the actual forces generated and applied to produce flakes during knapping. Our study introduces a novel solution to this problem in the form of an ergonomic hand-held synthetic hammerstone that can record the percussive forces that occur during knapping experiments. This hammerstone is composed of a deformable pneumatic 3D-printed chamber encased within a 3D-printed grip and a stone-milled striker. During knapping, hammer impact causes the pneumatic chamber to deform, which leads to a change in pressure that is measured by a sensor. Comparisons of recorded pressure data against corresponding force values measured using a force plate show that the synthetic hammer quantifies percussion forces with relatively high accuracy. The performance of this hammerstone was further validated by conducting anvil-supported knapping experiments on glass that resulted in a root mean square error of under 6%, while recording forces up to 730 N with successful flake detachments. These validation results indicate that accuracy was not sensitive to variations up to 15° from the vertical in the hammer striking angle. Our approach allows future studies to directly examine the role of percussive force during the stone knapping process and its relationship with both anatomical and technological changes during human evolution.

## Introduction

Tool use has been an important component of human evolution for the past ~3.3 million years [1–6]. Early stone tools were made by striking one stone against another to produce chips with sharp edges, which enabled hominins to obtain and pre-process food resources and potentially make tools out of other materials [7]. One approach to better understand how hominins made and used stone tools involves reconstructing the stone flaking process, known as ‘knapping’, by experimentally replicating the forms of various stone tools from the archaeological record [8, 9].

A critical aspect of stone knapping is that it requires the application of percussive force. To successfully detach flakes, knappers need to deliver percussive blows that transmit sufficient force to the core to initiate and sustain fracture propagation. Applying too little or too much force can result in undesirable outcomes, such as hinge/step terminations or platform crushing, features that negatively impact the subsequent knapping potential of the core [10, 11]. The ability to control the application of knapping force effectively and accurately has been shown to be intimately linked to manual skills and technical ‘know-hows’ [12–15], which may have been acquired among hominin toolmakers through cultural transmission mechanisms such as social learning, detailed copying, and active teaching [16]. In addition, how hominins controlled and applied percussive force would have been dictated by their functional anatomy, especially for the upper limb [17–19]. The hominin fossil record suggests that modern humans and Neandertals share a suite of hand morphology that is derived relative to that of earlier hominins and extant great apes, and these derived characteristics likely evolved by at least ~1.4 Ma [20, 21]. In contrast, the earliest hominin toolmakers appear to have retained various combinations of primitive, ape-like features in their hands and upper limbs [20, 22–24]. As such, clarifying the role of percussive force in the stone knapping process is critical for understanding the relationship between tool-making behaviour and the broader cognitive and anatomical changes that occurred during human evolution [25].

Laboratory tests have shown that the amount of percussive force required to detach a flake is directly related to factors such as the size or mass of the flake [26] and raw material [27–30]. Essentially, the larger the detached flake, the greater the force required [26, 31]. The ways in which force is transmitted to the core can further be varied by using different hammer materials, hammer striking angles, and striking speed [32, 33]. However, these studies of percussive force often rely on mechanical apparatuses to simulate the stone knapping process under highly controlled settings. Few studies to date have evaluated percussive force generated by human participants engaging in realistic knapping activities. For instance, several researchers [34–39] have used motion-sensing technology to calculate the potential energy and kinetic energy of hammer strikes based on parameters such as hammer mass, the velocity and trajectory length of the hammer during strikes, and the velocity of the wrist upon hammer impact. Bril et al. [35] observed that, while all participants applied greater kinetic energy when asked to detach larger flakes, experts were more energetically efficient and consistent at detaching flakes under different conditions (also see [39, 40]). Similarly, Nonaka et al. [38] showed that among the flakes detached by expert knappers, kinetic energy shares a significant positive relationship with flake length and area, while the kinetic energies delivered by novice and intermediate knappers are more variable and do not correlate with flake dimensions. However, a limitation of the kinematic approach is that the motion-sensing technology required can be logistically and financially expensive, and may not be easily accessible to many experimental knapping projects. More fundamentally, without direct measurements, it remains unclear how the estimated kinetic energies in these studies correspond to the actual percussive forces delivered upon hammer impact in these knapping trials.

To address this methodological issue, it is necessary to develop new sensorized devices that can directly gauge the amount of reaction forces experienced by the hammerstone during knapping activities. In this regard, a noteworthy study [41] constructed and utilized a sensorized hammerstone comprising a brass body in which load cells were encased. However, instead of positioning the load cells to gauge the percussive force at the point of impact, the load cells were placed to correspond to the locations of the thumb and the index finger in order to measure the reaction forces acting on the digits during simulated percussion activities [41]. One limitation of this study is that the triangular disc shape of the metal hammer does not resemble a typical hammerstone, and it remains unclear how effective this synthetic device can be used to detach flakes from brittle solids in actual stone knapping experiments. Thus, if we are to develop a device that can be flexibly incorporated into knapping experiments to measure percussive force, it not only needs an instrumental capacity for accurate measurements of reaction force upon impact, but also an ergonomic design that reasonably approximates the form of typical hammerstones.

Recent advances in sensor technology, such as customizable 3D printed pneumatic sensing chambers (PSC) [42, 43] may be leveraged to ameliorate these challenging issues. PSCs are soft and hollow structures that deform elastically when a force is applied, resulting in changes to the inner air volume that can then be measured. Tawk et al. [43] showed PSCs as novel, versatile, and low-cost fabrication systems that can detect and measure pressure changes using soft materials such as thermoplastic polyurethane (TPU) and thermoplastic elastomer (TPE). Subsequent research (e.g., [42]) extended the applicability of PSCs in the measurement of internal joint forces for the development of exosuits and exoskeletons. The primary advantages of this technology relevant to the stone knapping scenario is that the chamber itself is highly customisable and can withstand high forces relative to its size/weight.

Drawing on these advances, this study aims to develop a synthetic sensorized hammerstone with an embedded PSC to accurately measure percussive forces during knapping. Through this approach, we aim to provide an accessible tool that can be used to better understand the nature of forces during the striking phase of stone tool production. When used alongside other biomechanical methods, such as computer-based models, this device strengthens the ability to investigate the interconnection between force application, manual control and flake formation, opening up new research horizons to study the biomechanics of stone tool making among past hominins.

## Materials and methods

The process of creating a synthetic sensorized hammerstone suitable for measuring forces during stone knapping experiments involved a multidisciplinary approach, combining methods from archaeology, sensor development, and mechanics. The following describes the design process of the hammerstone, as well the characterization and application experiments.

### Sensorized hammerstone

**Fig 1:**
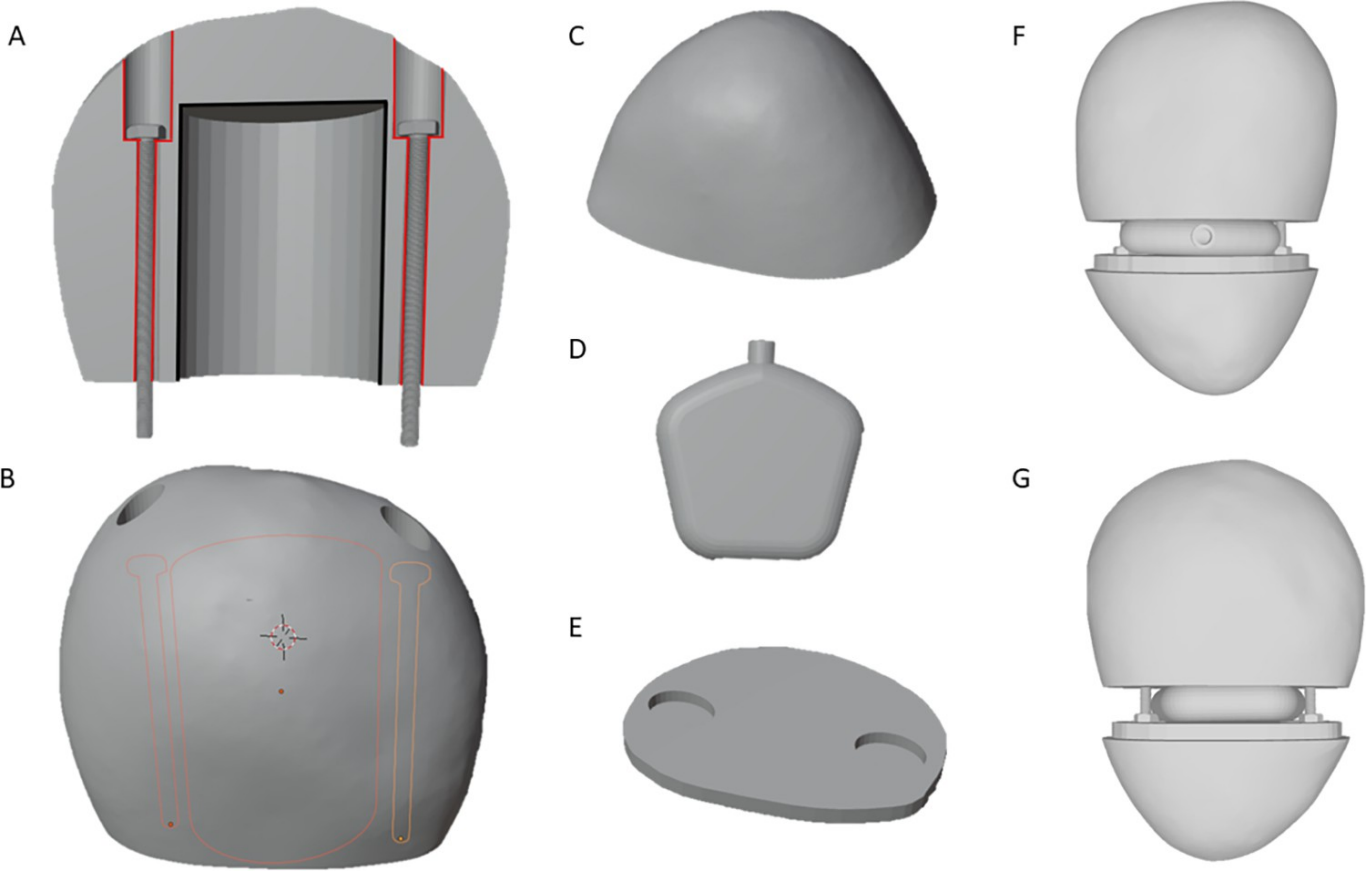
Components of the sensorized hammerstone. (A) Sectioned and (B) outside view of the 3D printed grip showing the internal channels used to secure the grip to the basalt stone striker (C). The void outlined in black in panel (A) was filled to increase the overall weight; (D) 3D printed pneumatic sensing chamber; (E) Platform for the connection of the parts. Panels (F) and (G) show front and rear views of the assembled hammerstone with the gripping part at the top, followed by the pneumatic chamber, the platform, and the stone striker.

To have consistent, repeatable results, a template was created for the hammerstone by selecting a rock with a shape and size suitable for comfortable gripping (Fig 1). The shape geometry of this rock was recorded using a high-resolution surface scanner Polyga Compact C210 (Polyga, Canada), and processed with FlexScan3D (Polyga, Canada) to smooth the surface and fill in small holes. The resulting 3D model was divided transversally into two distinct parts in Blender (3.1.2): the grip (Fig 1A and 1B) and the striker (Fig 1C). The gripping part was much larger than the striking part to ensure that when held in the hand, the fingers only contacted the gripping part. This design was essential to make sure that all forces from the striking part are transmitted through a deformable chamber (described below) that is wedged between the two parts. Additionally, the striker surface area was large enough to allow the knapper to produce flakes when striking the core.

### Design of the 3D printed grip

The gripping part of the hammerstone was reproduced using 3D printing in order to embed the channels and mechanism required for the force sensor. The brittle nature of most rocks was judged to be unsuitable for directly embedding these in the original material. The gripping part of the hammerstone was printed using fused deposition modelling 3D printing with the thermoplastic material, Polylactic Acid (PLA). As PLA density was much lower than that of the original rock, a pure PLA-based grip would create an imbalanced weight distribution in the hammerstone. To increase the total weight of the gripping part, a central void was created (outlined in black in Fig 1A) and filled with lead weights and resin. The weight of the PLA and filler material was carefully determined to match that of the original grip section of the rock (440 g).

Two longitudinal channels were designed to accommodate the M3 50 mm bolts that attach the gripping and striking parts. The top ends of the channels were recessed into the surface, such that the bolt heads did not protrude out when the stone was gripped in the hand. The channel and bolt lengths were chosen to be sufficiently long to allow for the PSC to be wedged between the grip and the striker, and as well to securely screw into the striker.

### Design of stone striker

The striking part is designated to be in contact with the core during the knapping process and was obtained by milling basalt rock. Basalt rock samples were first cut into a cube shape with a power-feed diamond saw, before being milled using a three-axes automated milling machine (Yubang YB4030P, Yubang Automation Control Equipment) to match the original scanned shape of the striking part of the hammerstone (Fig 1C). JDPaint 5.5 (Beijing Jingdiao Group) was used to convert the scanned shape of the striker into a 3D milling path. Following the milling process, a platform mirroring the contour of the base of the striker was 3D printed using PLA material (Fig 1E). This platform incorporated two countersunk profiles, facilitating the placement of nuts during assembly of the striking and gripping components. Subsequently, both the nuts and the platform underwent a sealing process, seamlessly bonded together using epoxy adhesive.

### Design of pneumatic sensing chamber

The PSC was designed to fit between the gripping and striking parts and shaped to accommodate the bolts holding two parts together (Fig 1D). With this arrangement, the PSC was subjected to a pistoning action when forces were applied to the striking part (e.g., by impacts on the core). The ability of the PSC to uniformly deform upon hammer impact was an important desired characteristic, and in addition to material choice, other important factors in this regard were the wall geometry and wall thickness. Based on previous results using similar setups [42, 43], a PSC of size 34.5 mm x 45.8 mm x 10 mm was designed that fit snugly between the striking and gripping parts of the hammerstone, as well as the bolts. The walls of the chamber were rounded with a 4 mm radius fillet to allow for easy deformation. On one of the longer side walls, a nozzle was designed to attach the pressure sensor tube.

**Fig 2:**
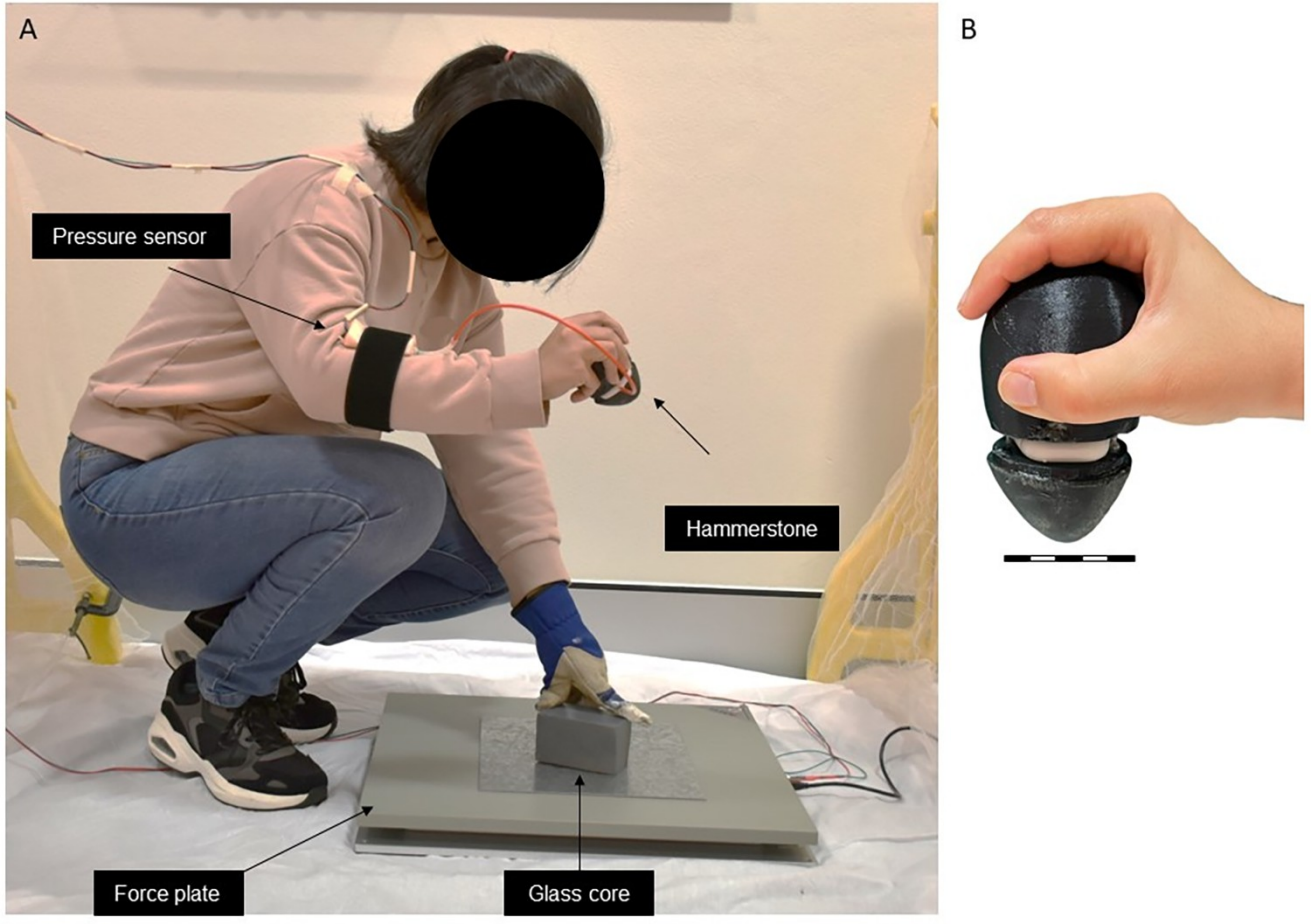
Overview of the experimental setup and the sensorized hammerstone. (A) Experimental setup with the glass core placed on the force plate. The user strikes the core with a sensorized hammerstone (illustrated in insets on the right) that is connected to a pressure sensor. (B) Close-up view of the hammerstone in the user’s grip. Scale bar is an approximation, as scale varies according to perspective.

PSC design was performed using Autodesk Inventor v2023 (Autodesk, United States) and the mesh exported as an STL file. To fabricate the PSC, we used FlashPrint v5 (Flashforge, China) to slice the STL file and it was printed on a Creator Pro 3D printer (Flashforge, China) using TPE as the material (PSC geometry and printing settings are provided in the S1 File and S1 Table). The fabricated PSC was connected to a 60 psi commercial pressure sensor (Honeywell International Inc., SSCDANN060PGAA5) via a flexible tubing. The pressure sensor signal was recorded on a digital acquisition device (NI DAQ, Model USB 6003) at 100 kHz. The addition of the flexible tubing and pressure sensor away from the hammerstone enabled the hammerstone to be held in the hand naturally with the DAQ and pressure sensor mounted further up the arm (Fig 2).

### Sensor characterization

To develop a model of the PSC characteristics, we conducted a series of tests that first calculated the maximum frequency response (bandwidth) of the PSC and then developed a data-driven model by mapping externally applied forces to PSC pressure changes.

### Bandwidth estimation

The purpose of the bandwidth estimation tests was to evaluate how quickly the PSC could react to an external force. PSC bandwidth was estimated using the procedure outlined in Alici et al. [44]. A mechanical impulse was applied on the chamber and the corresponding change in PSC pressure was recorded. This PSC signal was then compared to the response of a damped harmonic system, and the signal peak magnitudes as well as the time difference between peaks were used to compute the bandwidth. These tests were repeated 14 times, to get an average bandwidth of 405.23 Hz with a standard deviation of 11.1 Hz.

### Experimental setup

**Fig 3:**
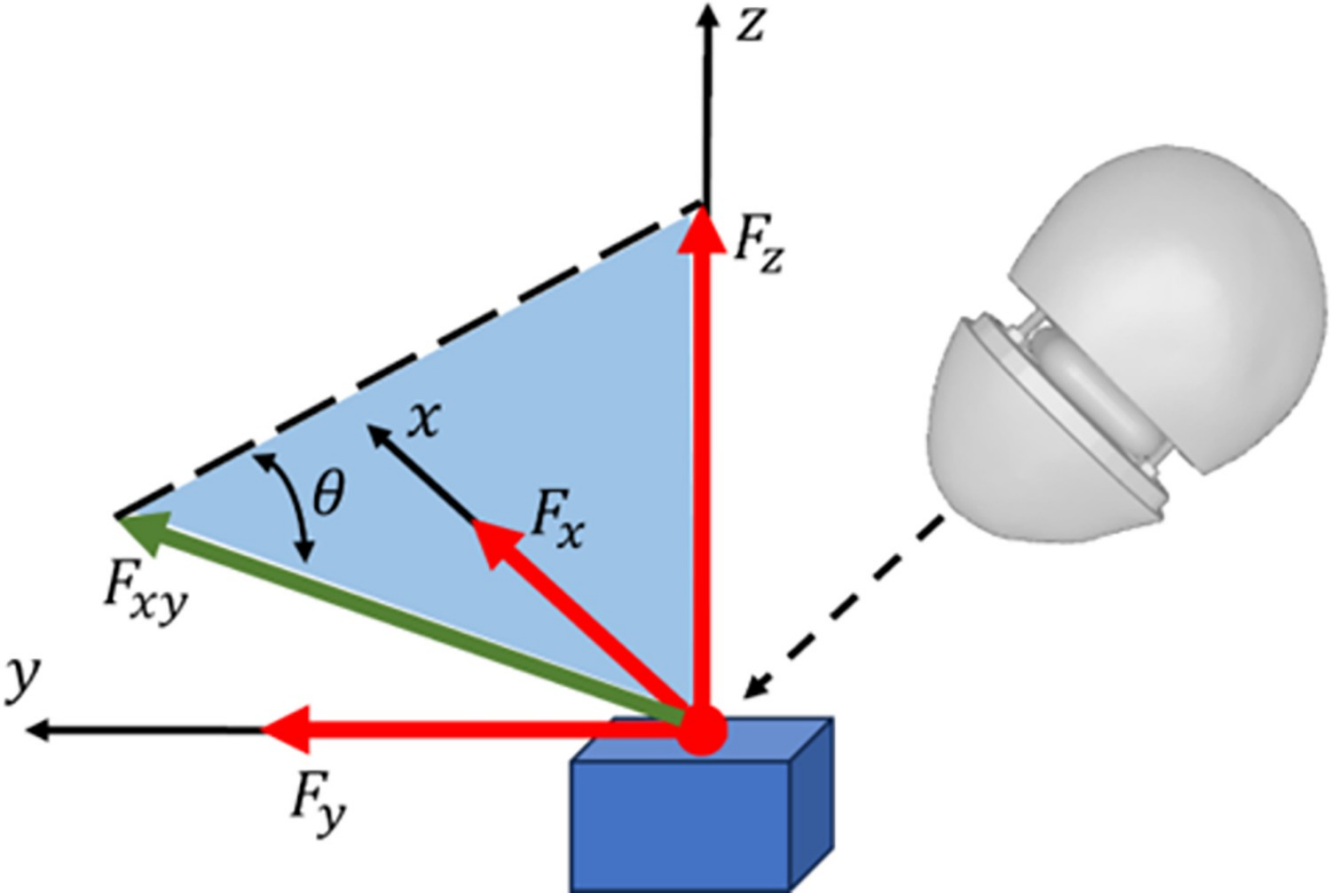
Calculation of strike angle θ from recorded force components at impact point.

We developed an experimental setup (Fig 2) using a force platform (Accugait, AMTI, USA) that recorded applied forces and moments along 3 orthogonal axes. Platform forces were recorded at 1 kHz and time synchronized with the pressure signal using a trigger TTL signal at the start of recording. Net force *F*_*n*_ was calculated as $F_{n}=\sqrt{F_{x}^{2}+F_{y}^{2}+F_{z}^{2}}$, where *F*_*x*_, *F*_*y*_, and *F*_*z*_ were the orthogonal force components recorded by the force plate (Fig 3). PSC pressure signals, recorded at 100 kHz, were low-pass filtered at 600 Hz using a zero-delay 2^nd^ order Butterworth filter to remove measurement noise. The directionality of the force (henceforth referred to as the strike angle) was calculated as the angle between the net horizontal force $F_{xy}=\sqrt{F_{x}^{2}+F_{y}^{2}}$ and the vertical force *F*_*z*_, as *θ* = tan^-1^ $\left(\frac{F_{z}}{F_{xy}}\right)$ (Fig 3). Note that *θ* = 90° implies a strike with the force applied exactly perpendicular to the core/force plate XY plane.

Note that the point of application of the force (usually the upper surface of the core), was displaced from the surface of the force platform. This resulted in additional moments being recorded by the force platform, however, the orthogonal forces recorded still reflected the true forces applied on the core. As we were only interested in characterising the force component in the present work, we discarded the moment recordings from further analysis.

Standardized core samples made of soda-lime glass were prepared with edges that had an exterior platform angle of 80° and placed on a rigid metal plate over the center of the force platform. We chose to use glass as the core materials because of the ease of fabrication of the core and the ability for glass to replicate other materials commonly used to make flakes [27, 45]. The core was struck repeatedly with the sensorized hammerstone over multiple trials with varying force magnitudes in a manner similar to anvil-supported knapping. Care was taken to replace cores, such that strikes occurred at fresh edges along the core. Each trial consisted of 10 strikes to the core and a total of 18 trials were recorded.

**Fig 4:**
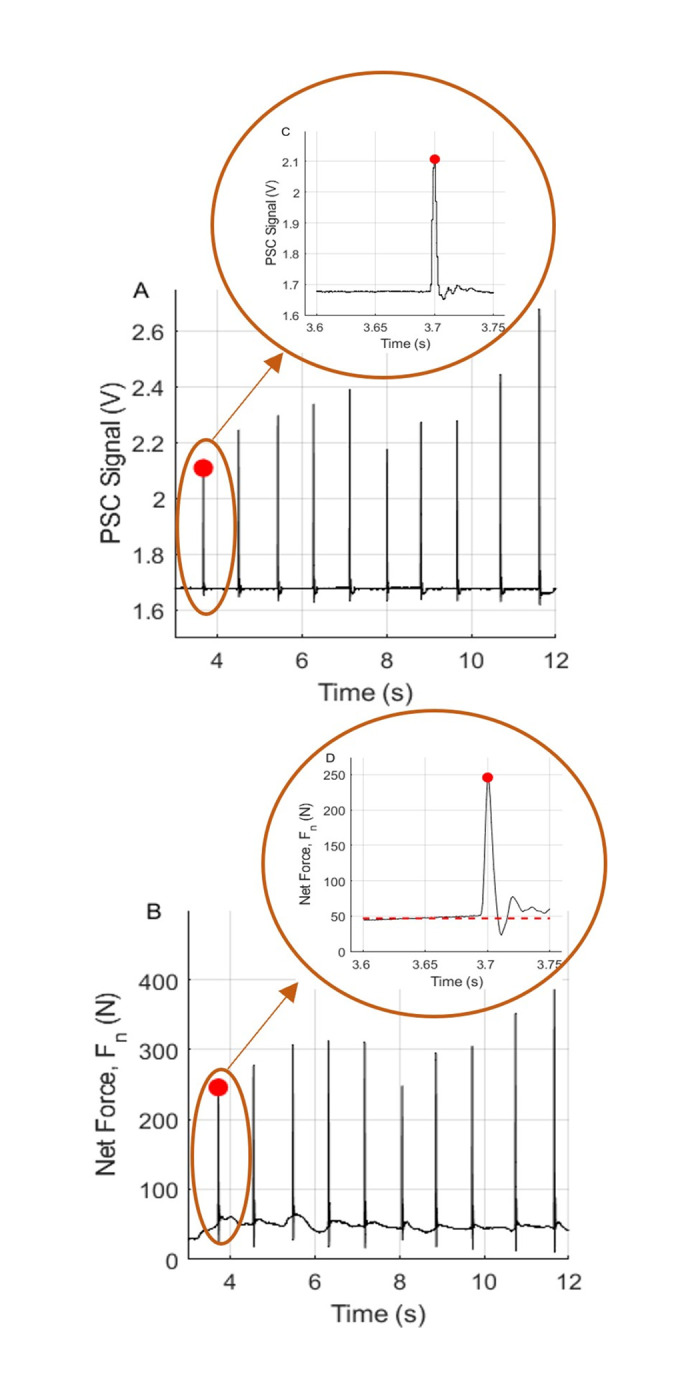
Analysis of hammerstone strikes, pressure signal, and force component. Panels (A) and (B) show the 10 strikes of the hammerstone resulting in peaks in the PSC pressure signal and net force, respectively. (C) and (D) show an expanded view of the first strike (peak circled in red on all panels). Dashed red line in panel (D) indicates the baseline force (due to the user resting their non-striking hand on the core) that was subtracted from the net force in post-processing.

Corresponding pairs of peak force (from the force plate) and pressure data (from the sensorized hammerstone) were identified using a custom-built MATLAB program in order to isolate the point of impact during each strike (Fig 4). The 10 highest peaks in each trial were then identified by detecting the points where the gradient of the signals crossed zero and the magnitude of the signals were high enough above a threshold (set manually). All trials were processed automatically in this manner to generate 180 sets of force and pressure data corresponding to the points of impact.

### Model fitting and validation

Experimental data were used to develop a mathematical model that mapped the applied net force *F*_*n*_ to the PSC pressure signal. For this model fitting stage, 16 of the 18 trials (i.e., 160 strikes) were used with the remaining 2 trials reserved for model validation. A linear regression model was fit to the 160 data points using MATLAB’s *fitlm* tool, with PSC pressure signal, *P*, as the independent variable and net force *F*_*n*_ as the dependent variable, resulting in the model, *F*_*n*_ = *aP*+*b*, with *a* = 381.02, and, *b* = −635.28.

**Fig 5:**
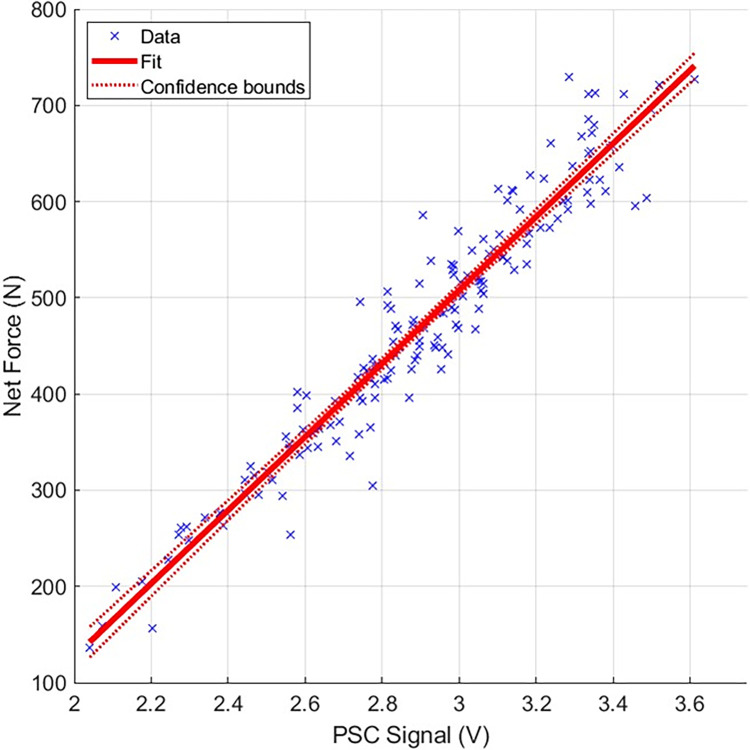
Linear regression model mapping PSC signal to net force. Data points indicate the extracted peaks at the point of impact.

The model fit shown in Fig 5, resulted in an adjusted *r*^2^ value of 0.92, and a root mean square error (RMSE) of 36.3 N. For context, the maximum force applied in the trials was ~730 N, which gives the model a % RMSE of 4.98%. Using a more complex model (e.g., a quadratic function) did not result in appreciable improvement in the RMSE nor in the *r*^2^ value. For the data used in model fitting, the strike angle ranged from 71.6° to 89.7° with a mean of 84.1°.

The model results were validated against the data from the two remaining trials (i.e., those not used in the fit). Using the linear model parameters, forces were estimated for the recorded PSC pressure signal and then compared against the actual force plate recorded forces. These comparisons resulted in a RMSE of 38.7 N (or ~5.6%) between the estimated and recorded forces.

### Application in knapping

From the characterization tests, peak strike force ranged from ~135 N to ~730 N across all trials. While these tests did result in flakes being produced, their primary purpose was to map the relationship between forces and the pressure signal. Consequently, an additional set of experiments was conducted where the focus was on testing the ability of the sensorized hammerstone to produce flakes from the glass core. An additional 6 trials (60 strikes) were recorded for this evaluation. The methodology for these experiments was identical to that described previously.

**Fig 6:**
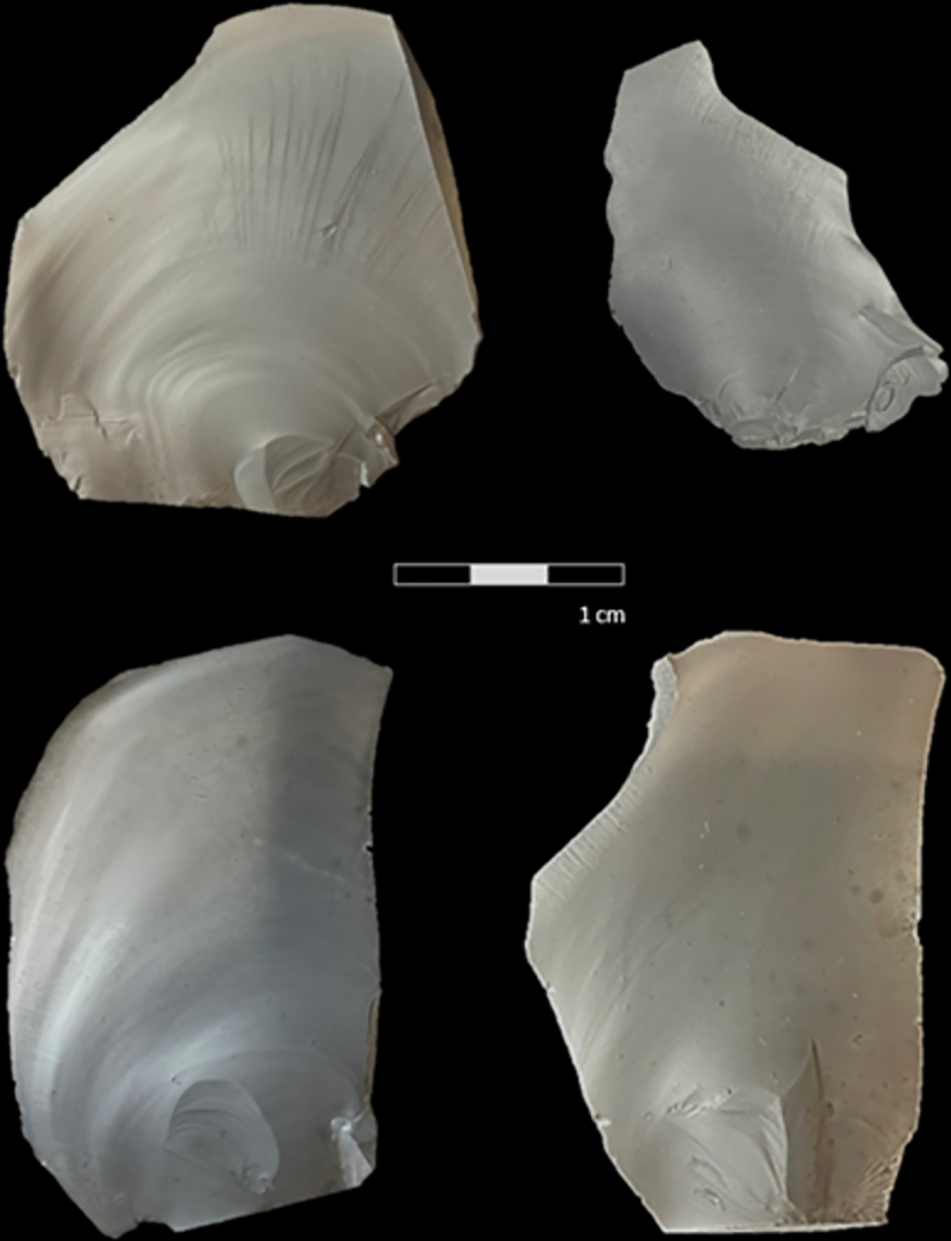
Ventral view of flakes produced during the trials. The flakes are positioned with the striking platform at the bottom. All flakes demonstrated typically expected features such as the striking platform, the bulb of percussion, ripple marks, and fissures.

The trials successfully produced sizable flakes that had a technological length ranging from 29.2 mm to 69.3 mm with typical conchoidal features that resemble those found in archaeological assemblages (Fig 6). Deviations in the angle of the force being applied were also measured to evaluate whether these affected the forces estimated from the model. Strike angle *θ*, ranged from 74° to 88.8° with a mean of 80.8° across all trials. Note that, using the definition of the angle of blow outlined by Dibble and Rezek [26] (see also [46]), these striking angles translate to angles of blow between 16° and 1.2° from the vertical with a mean of 9.2°. The forces produced in these trials were estimated using the linear model developed previously, and the error between the estimation and the actual force (as recorded by the force plate) was computed. No significant correlation between the force error and the strike angle was observed (correlation coefficient = 0.1, p = 0.44).

## Discussion

The prototype sensorized hammerstone proved to be effective in accurately recording the percussive forces (< 6% error) involved in the knapping process. This level of accuracy, while lower than commercial force transducers, agrees well with applications using similar technology [42, 43]. In the context of the forces developed during knapping (up to 730 N in our study), we assert that this accuracy is sufficient to analyse the mechanics of knapping. Notably, our design preserved the look, feel, and size of a typical hammerstone, allowing users to have an experience that closely approximates natural knapping settings while producing flakes. To the best of our knowledge, no such sensorized system exists with comparable efforts (e.g., [41] using non-ergonomically shaped hammerstones). We attribute the success of our design to incorporating state-of-the-art advances in 3D printing and stone milling as well as soft-sensor development, which in combination helped overcome the challenges posed by the reduced size and intricate shape of the hammerstone.

A key feature of our developed system is its portability. Once calibrated, the hammerstone may be used in non-laboratory settings, as well as in non-anvil based knapping styles such as freehand knapping. This capability eliminates the need for expensive equipment such as force plates and associated sensitive electronics, which are susceptible to damage when transported. Based on our experiments, the wired sensor attachment on the arm was unobtrusive, akin to wearing a watch, and allowed the user to freely move the arm while knapping. Future iterations could further miniaturize this component and incorporate wireless transmission of data to a computer or mobile device to allow full freedom of movement. Additionally, the hammerstone was cost-effective and easy to produce with all of the materials and components commonly available for purchase. The design concept was modular, allowing for differently shaped hammerstones (e.g., a more oblong-shaped hammerstone) and gripping (here we used an extended three-jaw chuck grip [47]) to be built with a similar sensing mechanism.

A potential concern regarding the reliability of the sensorized hammerstone is whether the measurement accuracy is affected by variation in the hammer striking angle. Modern knappers are known to adjust the striking angle, or the angle of blow [26], to facilitate flake detachment depending on core surface configurations and knapping gesture [10, 48]. The ability to apply suitable striking angles during knapping is, in fact, a key manual skill that distinguishes experts from novices [49–51]. Therefore, if the sensorized hammerstone is to be useful to future knapping experiments, then the device needs to be able to accommodate variable striking angles, at least to a level that can be reasonably expected during stone knapping. In our trials, the analysis indicated that the knapping force strike angle was about ±15° around the vertical. Notably, we found no correlation in the strike angle and the error between the model-estimated force and the actual recorded force. This result allows for some measure of flexibility in the usage of the hammerstone, with users able to vary the striking angle and not be constrained to perform perfect perpendicular (to the longitudinal axis of the hammerstone bolt mechanism) strikes on the core. However, it is important to note that, due to measurement difficulty, there is limited empirical quantitative data available on hammer striking angle associated with stone knapping activities (but see [31, 33]). While mechanical experiments have explored a range of angles, spanning up to 60° from the vertical with successful flake detachment [26, 31], the exact range of striking angles typically applied by knappers under different knapping conditions remains uncertain and warrants further investigation.

There were limitations to the application of our prototype device. First, in our experiments, we used glass as the core material and measured up to 730 N of strike force. It has been shown that glass and other glass-like raw materials, such as obsidian, require less force to detach flakes than do rock types such as chert and basalt [27]. While we did not intentionally limit the force being applied by the user (indeed our device could produce flakes well below the maximum recorded force), varying raw materials would likely yield force results that differ from those presented in this study. To address this, future development efforts should focus on enhancing the sensing range and conducting further trials to create a device capable of recording a wider range of forces, potentially requiring a stronger pneumatic chamber. The potential challenge here lies in the possibility that non-linearity and viscosity of the pneumatic chamber material may play larger roles at very high dynamic forces. Second, while the data accuracy of our device is relatively high when the hammer striking angle is ±15° around the vertical, it is likely that the force estimating accuracy deteriorates for highly oblique strikes (i.e., more than 15° off the vertical). One possible solution would be to replace the mono-chamber design with a multi-directional chamber capable of measuring orthogonal forces (e.g., [52]). This is a promising avenue for future research but also presents challenges due to the limited space available inside the hammerstone. Additionally, it is likely that the elastic deformation of the pneumatic chamber affected the profile of the impact force of the hammerstone on the core (when compared to say a solid stone hammer). To an extent, any sensor placed at the interface between the striker and the hand grip will affect the impact force. In future iterations we can minimize this effect by exploring the use of stiffer sensors (that result in smaller deformations) at this interface. Finally, our design requires access to some specialised equipment such as 3D printers, a stone-milling machine, and a force plate (or similar calibration device). Both 3D printing and stone-milling technology have become increasingly accessible, and many cost-effective solutions are currently available. While force plates are relatively expensive, it should be noted that any sensor developed will only be as good as the device used to calibrate it, and that this is a reasonable trade-off for the ability to record forces during knapping.

Our work, for the first time, demonstrates a versatile method that can directly measure percussive force during stone knapping experiments. The novel approach opens up avenues of research that could provide insights into a range of important research topics relating to stone tool technology and human evolution. For one, it offers a means to systematically investigate the role of force application in flake formation, especially how force as an independent variable may affect variability in flake attributes such as dimension and the size of the bulb of percussion. Importantly, the percussive force measured in previous mechanical flaking experiments [26, 33] is better described as a dependent variable, as the parameter was not directly controlled and instead was contingent on other knapping parameters, such as platform configurations and the angle of blow [31] (see similar discussion about platform width in [53]). With our new device, it is now possible to independently manipulate hammer striking force during flaking irrespective of core and platform morphology. Clarifying the relationship between percussive force and flake variation has strong potential to improve our ability to infer force application practices by early hominin toolmakers on the basis of archaeological stone tool variability.

The ability to record knapping forces can also be applied to expand current understanding of stone knapping biomechanics. There is great interest in investigating body kinematics and the pressure and muscle recruitments that occur during stone tool production [19, 54–58]. For instance, several studies have shown that stone knapping incurs substantial pressure and muscle recruitment in the hand and digits of both the dominant hand (that delivers the hammer blow) and the non-dominant hand (that holds or stabilises the core) [19, 55, 56, 59–64]. With the sensorized hammerstone presented here, there is now the additional capacity to gauge percussive forces associated with kinematic and pressure data during knapping activities. For instance, the use of this device in conjunction with pressure sensor pads, such as those used by Williams et al. [19, 54] and Williams-Hatala et al. [59], could help quantify the relationship between net external force and pressure distribution at the interface between the hammerstone and the digits. Furthermore, coupling these data with biomechanical analysis using state-of-the-art computer models (e.g., [65–67]) will help better understand the propagation of forces through the musculoskeletal structures of the human body during stone tool production. Computer-based musculoskeletal models have already proven to be an invaluable tool for comparative functional morphology in human and nonhuman primates. These models have been particularly impactful in examining the dynamics and energetics influencing the evolution of bipedalism in hominins [68, 69] and exploring changes in hominin hand capabilities [70, 71]. In this context, our device facilitates the integration of force measurements under realistic knapping experiments. This enhanced capability is expected to enrich computer models, a growing area of interest both in Anthropology and Archaeology, providing invaluable new insights into the dynamics of stone knapping and its correlation with significant anatomical developments throughout the past ~3 million years of human evolution.

## Supporting information

S1 File 3D geometry of the pneumatic chamber. (STL)

S2 File 3D geometry of the gripping part. (STL)

S3 File 3D geometry of the stone striker. (STL)

S4 File 3D geometry of the platform. (STL)

S5 File Pressure and force data from the model and validation. (TXT)

S1 Table Printing settings of the pneumatic chamber. (PDF)

S2 Table Printing settings of the gripping part. (PDF)
