# Supplementary material for: Design and development of a sensorized hammerstone for accurate force measurement in stone knapping experiments
Source: PLoS One. 2024 Sep 17;19(9):e0310520. doi: 10.1371/journal.pone.0310520 (PMC11407656; doi:10.1371/journal.pone.0310520)
Supplement: S2 Table — (PDF) [file pone.0310520.s007.pdf]

S2. Table. 3D printing settings for gripping part

Printer used: Creator Pro Inventor

Material used: PLA+, eSun

Parameter	Value	Unit
Temperature		
Extruder temperature	220	°C
Bed temperature	60	°C
Layer		
Layer Height	0.18	mm
First Layer Height	0.27	mm
Speed		
Base Print Speed	20	mm/s
Travel Speed	70	mm/s
Minimum Speed	5	mm/s
First Layer Max Speed	10	mm/s
Retraction		
Extruder Length	1.3	mm
Retract Speed	30	mm/s
Extrude Speed	30	mm/s
Shells		
Shell counts	10	
Overlap Perimeter	30	%
Speed		
Exterior Speed	70	%
Exterior Maximun Speed	80	mm/s
Infills		
Top Solid Layers	10	
Bottom Solid Layers	10	
Fill Density	100	%
Fill Pattern	Hexagon	
Overlap Perimeter	15	%
Vase Mode	Disabled	
